# Limited sequence polymorphisms of four transmission-blocking vaccine candidate antigens in *Plasmodium vivax* Korean isolates

**DOI:** 10.1186/1475-2875-12-144

**Published:** 2013-04-30

**Authors:** Jung-Mi Kang, Hye-Lim Ju, Sung-Ung Moon, Pyo-Yun Cho, Young-Yil Bahk, Woon-Mok Sohn, Yun-Kyu Park, Seok Ho Cha, Tong-Soo Kim, Byoung-Kuk Na

**Affiliations:** 1 Department of Parasitology and Institute of Health Sciences, Gyeongsang National University School of Medicine, Jinju, 660-751, South Korea; 2 Department of Pathology, College of Medicine, Korea University, Seoul, 136-705, South Korea; 3 Department of Parasitology and Inha Research Institute for Medical Sciences, Inha University School of Medicine, Incheon, 400-712, South Korea; 4 Department of Biotechnology, College of Biomedical and Health Sciences, Konkuk University, Chungju, 380-701, South Korea

**Keywords:** *Plasmodium vivax*, Transmission-blocking vaccine, Genetic polymorphism, Korea

## Abstract

**Background:**

Transmission-blocking vaccines (TBVs), which target the sexual stages of malaria parasites to interfere with and/or inhibit the parasite’s development within mosquitoes, have been regarded as promising targets for disrupting the malaria transmission cycle. In this study, genetic diversity of four TBV candidate antigens, Pvs25, Pvs28, Pvs48/45, and PvWARP, among *Plasmodium vivax* Korean isolates was analysed.

**Methods:**

A total of 86 *P. vivax-*infected blood samples collected from patients in Korea were used for analyses. Each of the full-length genes encoding four TBV candidate antigens, Pvs25, Pvs28, Pvs48/45, and PvWARP, were amplified by PCR, cloned into T&A vector, and then sequenced. Polymorphic characteristics of the genes were analysed using the DNASTAR, MEGA4, and DnaSP programs.

**Results:**

Polymorphism analyses of the 86 Korean *P. vivax* isolates revealed two distinct haplotypes in Pvs25 and Pvs48/45, and three different haplotypes in PvWARP. In contrast, Pvs28 showed only a single haplotype. Most of the nucleotide substitutions and amino acid changes identified in all four TBV candidate antigens were commonly found in *P. vivax* isolates from other geographic areas. The overall nucleotide diversities of the TBV candidates were much lower than those of blood stage antigens.

**Conclusions:**

Limited sequence polymorphisms of TBV candidate antigens were identified in the Korean *P. vivax* population. These results provide baseline information for developing an effective TBV based on these antigens, and offer great promise for applications of a TBV against *P. vivax* infection in regions where the parasite is most prevalent.

## Background

Malaria is one of the most important infectious diseases and remains a significant global public health problem [1]. Although extensive studies have aimed to inform the development of an effective malaria vaccine, no licensed vaccine against malaria has yet emerged. A number of potential malaria vaccine candidates are under various stages of clinical development [2], but their antigenic diversity among clinical isolates is one of the major obstacles in the design of an effective malaria vaccine. Therefore, understanding the epidemiology of the parasite and the genetic polymorphisms of vaccine candidate antigens in worldwide clinical isolates is critically important for the successful development of a malaria vaccine.

Transmission-blocking vaccines (TBVs), which target the sexual stages of malaria parasites to interfere with and/or block the parasite’s development within mosquitoes, have been regarded as important targets for disrupting the malaria transmission cycle [3-5]. In particular, TBVs have been considered as a promising strategy for controlling *Plasmodium vivax*, since this parasite has a unique biological characteristic in which it undergoes early gametocytogenesis prior to the manifestation of clinical symptoms, which enables parasite transmission prior to treatment. To date, several sexual stage antigens with potent transmission blocking activities have been identified and characterized. These include the pre-fertilization antigen, Pvs48/45, and the post-fertilization antigens, Pvs25 and Pvs28. Pvs25 and Pvs28, the most extensively studied TBV candidate antigens of *P. vivax*, are specifically expressed on the surfaces of the zygotes and ookinetes of the malaria parasite [6], and play an essential role in both the survival of ookinetes in the mosquito midgut and in the subsequent penetration of the midgut epithelium and transformation into oocysts [7]. These proteins have been shown to exhibit strong immunogenicities and potent transmission blocking activities [8-13], thus supporting their potential for TBVs. More recently, several other *P. vivax* proteins have been identified and partially characterized as potential antigens for a mosquito-stage TBV, including Pvs230 [14,15], chitinase (PvCHT1) [16,17], the circumsporozoite thrombospondin-related anonymous protein-related protein (PvCTRP) [18], and the von Willebrand factor A domain-related protein (PvWARP) [19,20]. However, genetic polymorphism in these vaccine candidate antigens can hamper the efficacy of a vaccine. Therefore, identifying genetic variations in sexual stage antigen genes among the *P. vivax* population is an important task for designing effective anti-malarial control measures.

In this study, genetic variations of four TBV candidate antigens, Pvs25, Pvs28, Pvs48/45, and PvWARP, in *P. vivax* Korean isolates were analysed. These TBV candidate antigens showed highly limited sequence polymorphisms and lower nucleotide diversities compared to those observed in blood stage antigens. These results collectively support the potential of the antigens as promising targets for *P. vivax* TBV vaccine development.

## Methods

### Blood samples

A total of 86 blood samples, which were collected from Korean patients infected with *P. vivax* in Korea between 1999 and 2010, were used in this study [21]. *Plasmodium vivax* infection was confirmed by microscopic examination of thin blood smears and polymerase chain reaction (PCR) [22]. All patients had a typical febrile illness and had not been abroad, especially where malaria is endemic, for at least two years when their blood samples were collected. Approximately 5 ml of blood was collected from each individual, which was separated into packed cells and plasma and stored at −80°C until use. Blood collections were conducted after obtaining informed consent from the patients and by adhering to the institutional ethical guidelines reviewed and approved by either the Ethics Committee of Gachon University of Medicine and Science or the Inha University School of Medicine.

### Genomic DNA extraction and amplification of TBV candidate antigens

Genomic DNA was extracted from 200 μl of each blood sample using a QIAamp Blood Kit (Qiagen, Valencia, CA, USA). Amplification of TBV candidate antigens was performed with PCR by using specific primers for each antigen (Table 1). These primers were designed to amplify full-length gene of each antigen. The amplification reactions for Pvs25, Pvs28, and PvWARP were performed using the following thermal cycling conditions: 94°C for 5 min, 30 cycles at 94°C for 1 min, 52°C for 1 min, and 72°C for 1 min, followed by a final extension at 72°C for 10 min. Amplification of Pvs48/45 was conducted with the following thermal cycling conditions: 94°C for 5 min, 30 cycles at 94°C for 1 min, 55°C for 1 min, and 72°C for 1.5 min, followed by a final extension at 72°C for 10 min. In order to reduce the likelihood of possible nucleotide misincorporations, Ex Taq DNA polymerase (Takara, Otsu, Japan) with a proof-reading function was used in all the PCR reactions. The PCR product was analysed on a 1.2% agarose gel, purified from the gel, and then ligated into the T&A cloning vector (Real Biotech Corporation, Banqiao City, Taiwan). Each ligation mixture was transformed into *Escherichia coli* DH5α competent cells, and positive clones with the appropriate insert were selected by colony PCR. The nucleotide sequences of the cloned inserts were analysed by automatic DNA sequencing. In order to verify the sequences, at least three clones from each isolate were sequenced in both directions. Some isolates underwent two or three-fold sequence coverage to confirm the presence of rare polymorphisms. The nucleotide sequences reported in this study have been deposited in the GenBank database under the accession numbers JX667760–JX667767 (Pvs25, JX667760–JX667761; Pvs28, JX667762; Pvs48/45, JX667763–JX667765; PvWARP, JX667766–JX667767).

**Table 1:** Oligonucleotide primers used in this study

	**Primer name**	**Sequences**
Pvs25	Pvs25 F	5′-ACCATCCGAGCGGAAAGGAAC-3′
	Pvs25 R	5′-GTCGGTAAGTTCAGTAAAGAA-3′
Pvs28	Pvs28 F	5′-CGATTCCCCCTCCCCACTTTT-3′
	Pvs28 R	5′-GTGTATGTTTGTGTGTGTGTG-3′
Pvs48/45	Pvs48/45 F	5′-ATGTTGAAGCGCCAGCTCGCCAACC-3′
	Pvs48/45 R	5′-TCAGAAGTACAACAGGAGGAGCACAAT-3′
PvWARP	PvWARP F	5′-ATGAAAGGCGCACACGCCGTGTCC-3′
	PvWARP R	5′-TCAGTCCGTAGAGTCGCTGTCCCC-3′

### Sequence polymorphism analyses

Nucleotide and deduced amino acid sequences of each gene were analysed using EditSeq and SeqMan in the DNASTAR package version 4.0 (DNASTAR, Madison, WI, USA). DNA sequence polymorphism analyses were performed on all 86 sequences of each TBV antigen. The number of segregating sites (S), haplotype diversity (Hd), nucleotide diversity (π), and the average number of pair-wise nucleotide differences within the population (*K*) were calculated using the DnaSP ver. 5.10.00 package [23]. The numbers of synonymous (dS) and non-synonymous (dN) substitutions were estimated and compared by the Z-test (P < 0.05) in the program MEGA4 [24], using the Nei and Gojobori’s method [25] with the Jukes and Cantor correction. The standard error was determined by 1,000 bootstrap replications.

## Results and discussion

*Plasmodium vivax* re-emerged in South Korea in 1993 and remains a significant public health problem in the country. The outbreak has continued with fluctuating numbers of annual indigenous cases since the re-emergence, with total accumulated cases of up to 32,000, although the number of annual cases is currently decreasing [26]. Several recent studies have strongly suggested that the genetic diversity of *P. vivax* Korean isolates has rapidly disseminated in recent years [21,27-29], and that local transmission of the parasite is possibly established in South Korea. Therefore, understanding the nature of genetic polymorphism of *P. vivax* circulating in South Korea will be helpful for understanding the nationwide parasite heterogeneity and for the implementation of malaria control programes in the country, as well as for the development of an effective vaccine. In this study, the genetic diversity of four TBV candidate antigens, Pvs25, Pvs28, Pvs48/45, and PvWARP, were analysed.

**Figure 1:**
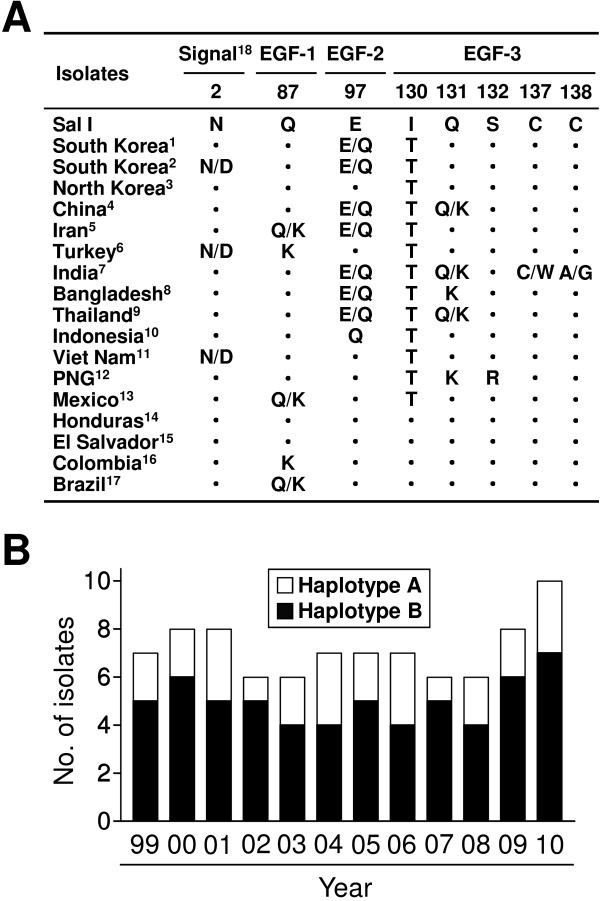
**Polymorphism analysis of Pvs25 in *Plasmodium vivax *Korean isolates.** (**A**) Distribution of the most commonly identified amino acid variants in Pvs25 among global isolates of *Plasmodium vivax*. Dot represent identical amino acid residue compared to Sal I. ^1^[This study], ^2^[37], ^3^[32], ^4^[35], ^5^[33], ^6^[ABG29073], ^7^[36], ^8^[31], ^9^[9], ^10^[32], ^11^[ABG29072], ^12^[31], ^13^[34], ^14^[32], ^15^[32], ^16^[32], ^17^[32], ^18^Signal peptide region. (**B**) Annual distribution of Pvs25 haplotypes during the study period, 1999–2010. The 86 Pvs25 sequences from Korean isolates were analysed as per the year of collection.

The gene encoding Pvs25 was successfully amplified from the 86 *P. vivax* Korean isolates. Compared to the Sal I sequence, the 86 Pvs25 sequences showed polymorphisms at three nucleotide sites (G289C, T389C, and T639A), of which two nucleotide substitutions resulted in amino acid changes (E97Q and I130T). One nucleotide substitution (T639A) resulted in a synonymous amino acid substitution and this nucleotide substitution was conserved in all 86 sequences. Pvs25 consists of three parts, which are characterized by an N-terminal signal peptide sequence followed by four epidermal growth factor (EGF)-like domains and a glycosylphosphatidylinositol (GPI) anchor [6]. The amino acid substitution E97Q was located in the second EGF-like domain (EGF-2), while the I130T substitution was found in the third EGF-like domain (EGF-3) (Figure 1A). These amino acid changes classified the Korean isolates into two different haplotypes; haplotypes A and B. The E97Q substitution was found in only haplotype A, while the I130T substitution was conserved in both haplotypes. Compared to previously reported Pvs25 sequences from worldwide *P. vivax* isolates, the amino acid substitutions found in the Korean isolates were commonly identified in isolates from other Asian countries and no novel amino acid changes were identified. Haplotype B was more prevalent (69.8%) than haplotype A (30.2%) and no difference was found in the annual prevalence of these haplotypes (Figure 1B).

In comparison with the Sal I sequence, the 86 Pvs28 sequences showed nucleotide polymorphisms at three positions, A154C, T346C, and C419G, which were evenly distributed in all 86 sequences, resulting in only one haplotype (haplotype 1). Two nucleotide substitutions (A154C and C419G) resulted in non-synonymous amino acid substitutions (M52L and T140S), and the other (T346C) revealed a synonymous amino acid substitution (Table 2). Pvs28 has a conserved structure, which consists of an N-terminal signal peptide sequence, four EGF domains, tandem repeat sequences (Gly-Ser-Gly-Gly-Gln/Asn; GSGGE/D), and a GPI anchor [6]. Amino acid substitutions M52L and T140S were identified in the EGF-1 domain and the EGF-3 domain, respectively. The two amino acid changes found in the 86 Pvs28 sequences were commonly identified in sequences from isolates from different countries. All of the 86 Pvs28 sequences contained seven copies of the GSGGE/D tandem repeat at the end of the EGF-4 domain.

**Table 2:** **Amino acid variations identified in Pvs28 of *Plasmodium vivax *Korean isolates**

**Isolates**	**Signal**^**9**^	**EGF-1**		**EGF-2**				**EGF-3**			**EGF-4**		**THR**^**11**^
	**5**	**52**	**53**	**65**	**98**	**105**	**106**	**110**	**116**	**140**	**159**	**Repeat**^**10**^	**224**
Sal I	H	M	A	T	L	E	V	Y	L	T	K	6	I
South Korea^1^	•	L	•	•	•	•	•	•	•	S	•	7	•
South Korea^2^	•	L	•	•	•	•	•	•	•	S	•	6	•
China^3^	•	M/L	•	•	L/I	E/K	•	•	L/V	T/S	•	5−6	I/M
Iran^4^	•	L	•	T/K	•	•	•	•	•	S	•	4−6	•
Bangladesh^5^	•	M/L	•	T/K	L/I	•	V/E	N	L/V	T/S	K/R	5−7	I/M
India^6^	H/Y/T	M/L	A/V	T/K	L/I	E/K	•	•	L/V	T/S	•	3−6	I/M
Thailand^7^	•	M/L	A/V	T/K	L/I	E/K	V/E	N	L/V	T/S	•	5−7	•
Mexico^8^	•	L	•	•	•	•	•	•	•	S	•	5−6	•

^1^[This study], ^2^[37], ^3^[35], ^4^[33], ^5^[31], ^6^[36], ^7^[9], ^8^[34], ^9^Signal peptide region, ^10^the copy number of the GSGGE/D tandem repeats; ^11^the C terminal hydrophobic region. Dot represent identical amino acid residue compared to Sal I.

Pvs25 and Pvs28 are the most advanced TBV candidates [10,11,30], which show limited polymorphisms among worldwide *P. vivax* isolates and the polymorphisms present in clinical isolates do not appear to hamper the efficacy of the candidate antigens as TBVs [8-13]. The amino acid changes found in Pvs25 and Pvs28 among the Korean isolates were not novel, but are commonly identified in previously studied worldwide *P. vivax* isolates [9,31-36]. Interestingly, the overall genetic polymorphisms of the 86 Pvs25 and Pvs28 sequences were more limited than those previously analysed in Korean isolates [37]. With respect to Pvs25, the main amino acid substitutions, E97Q and I130T, were commonly identified in the samples analysed in this study and in the previous report. However, several minor amino acid substitutions that were found in very low frequencies in a small number of isolates in the previous study [37] were not identified in this study. With respect to Pvs28, the minor amino acid changes in the tandem repeat region of the EGF-4 domain observed in the previous study [37] were not found in the 86 sequences analysed in the current study. These results collectively suggest that genetic polymorphism of the Pvs25 and Pvs28 genes in Korean *P. vivax* isolates is more limited than previously presumed.

**Figure 2:**
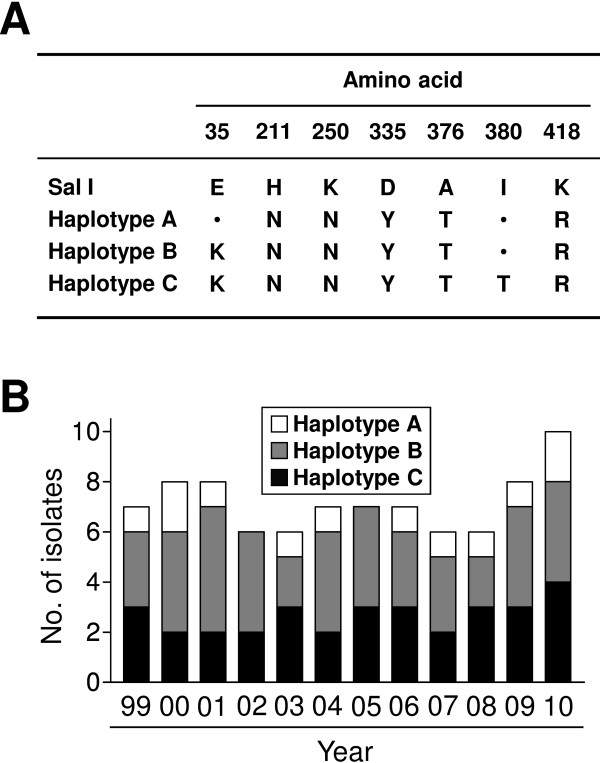
**Polymorphism analysis of Pvs48/45 in *Plasmodium vivax *Korean isolates.** (**A**) Comparison of amino acid variants between three haplotypes of Pvs48 Korean *Plasmodium vivax* and Sal I. Dot represent identical amino acid residue compared to Sal I. (**B**) Annual distribution of Pvs48/45 haplotypes during the study period, 1999–2010. The 86 Pvs48/45 sequences from Korean isolates were analysed by year of collection.

Pvs48/45 is a homologous protein with Pfs48/45, which is expressed on gametocytes and gametes of *Plasmodium falciparum* and appears to be involved in the fertilization process and zygote formation [38]. Because antibodies against Pfs48/45 inhibit infection and development of *P. falciparum* in mosquitoes at an early stage, presumably by inhibiting parasite fertilization, it is thus regarded as a TBV candidate antigen [39]. Pvs48/45 is still awaiting pre-clinical study, but its potential as a target of TBV has been addressed previously [40]. A total of 86 Pvs48/45 sequences were successfully obtained from the 86 *P. vivax* Korean isolates. Compare to the Sal I sequence, the 86 Pvs48/45 sequences showed nucleotide polymorphism at seven sites (G103A, C631A, C750A, G1003T, G1126A, T1139C, and A1235G), which resulted in dimorphic polymorphisms at seven amino acid positions (E35K, H211N, K250N, D335Y, A376T, I380T, and K418R). No synonymous nucleotide polymorphism was identified in the sequences. Based on these amino acid substitutions, Pvs48/45 of the Korean isolates were classified into three different haplotypes; haplotypes A, B, and C (Figure 2A). The five amino acid substitutions, H211N, K250N, D355Y, A376T, and K418R, were conserved in all 86 Pvs48/45 sequences. The E35K substitution was found in haplotypes B and C, while the I380T amino acid change was found in only haplotype C. The frequency and annual distribution of the Pvs48/45 haplotypes revealed that haplotype A (37.2%) and haplotype B (48.8%) were prevalent and their frequencies did not vary significantly across years (Figure 2B).

**Figure 3:**
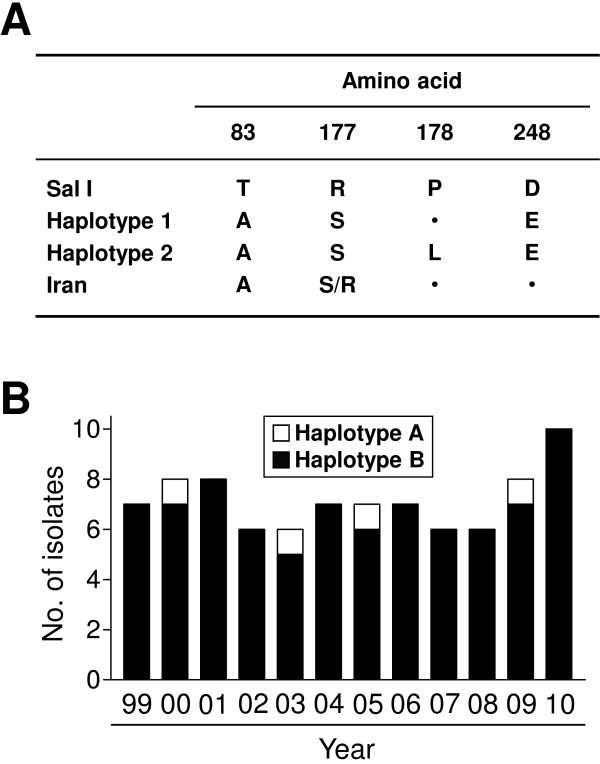
**Polymorphism analysis of PvWARP in *Plasmodium vivax *Korean isolates.** (**A**) Comparison of amino acid variants between two haplotypes of PvWARP Korean *Plasmodium vivax* and Iranian isolates [44]. Dot represent identical amino acid residue compared to Sal I. (**B**) Annual distribution of PvWARP haplotypes during the study period, 1999–2010. The 86 PvWARP sequences from Korean isolates were analysed by year of collection.

WARP is a highly conserved, soluble protein that is expressed in the late ookinetes and early oocysts of malaria parasites [19,41]. The protein mediates ookinete attachment to the mosquito midgut, differentiation of ookinetes to oocysts, and interactions with the mosquito basal lamina. Passive immunization of an anti-WARP antibody significantly reduced *Plasmodium* development, which suggested its potential as a TBV candidate antigen [42,43]. *Plasmodium falciparum* WARP (PfWARP) is known to show limited polymorphism in clinical isolates [43]. PvWARP also showed high sequence conservation among clinical *P. vivax* isolates from Iran [44]. Sequence analysis of the 86 PvWARP sequences revealed a total of four nucleotide substitutions (A247G, A531T, C533T, and C744A) compared to the Sal I sequence. These nucleotide substitutions resulted in non-synonymous amino acid changes (T83A, R177S, P178L, and D248E) (Figure 3A). Two of the amino acid substitutions (T83A and R177S) were previously reported from *P. vivax* isolates collected in Iran [44], but the other changes (P178L and D248E) have not been reported previously. A sequence analysis of the deduced amino acid sequences classified the 86 PvWARP into two different haplotypes (haplotypes 1 and 2), in which the P178L amino acid substitution was identified only in haplotype 2, while the other three amino acid changes were conserved in both the haplotypes. Haplotype 2 was predominant (95.3%) in all tested years and only four isolates of haplotype 1 were found in 2000, 2003, 2005, and 2009 (Figure 3B).

One of the major obstacles in the development of an effective malaria vaccine is the genetic polymorphism of genes encoding potential vaccine candidate antigens in natural parasite populations. Several studies have shown that TBV candidates have limited polymorphism compared to blood stage vaccine candidate antigens [14,33,34,45]. Since blood stage antigens are targets of host antibody responses, it is thereby likely that they would evolve high genetic diversity to increase the parasite’s immune evasion. Analysis of nucleotide diversity and genetic differentiation of four TBV candidate antigens of the 86 Korean isolates also revealed that limited genetic diversity was identified in Korean *P. vivax* isolates. The average number of pair-wise nucleotide differences (*K*) for Pvs25, Pvs28, Pvs48/45, and PvWARP was 0.427, 0.000, 0.716, and 0.090, respectively (Table 3). The overall haplotype diversity (Hd) was 0.427 ± 0.040, 0.000, 0.611 ± 0.026, and 0.090 ± 0.041 for Pvs25, Pvs28, Pvs48/45, and PvWARP, respectively (Table 3). As expected, the overall nucleotide diversities of the four TBV candidates, Pvs25 (π = 0.00065), Pvs28 (π = 0.00000), Pvs48/45 (π = 0.00053), and PvWARP (π = 0.00010), were much lower than those of blood stage proteins such as the C-terminal 42 kDa region of the merozoite surface protein-1 (PvMSP-1_42_; π = 0.01586) [21] and the Duffy binding protein (PvDBP; π = 0.00299) [46], which were determined from the same *P. vivax* isolates used in this study. Previous studies have suggested that the antigenic variation of Pvs25 is more limited than that of Pvs28 [6,35]; however, in the Korean isolates, the nucleotide diversity of Pvs28 was lower than that of Pvs25, as only a single haplotype of Pvs28 was identified. Polymorphism analyses of several blood stage antigens, including MSP-1, MSP-3α, and DBP, have strongly suggest that the genetic diversity of Korean *P. vivax* population has rapidly disseminated in recent years [21,27-29]. However, in the present study, there was no evidence of genetic discrimination in any of the TBV candidates during the tested years, since no significant correlations were identified between the haplotypes and their annual distributions. Balancing selection has been inferred to maintain high levels of polymorphism in PvMSP-1_42_[21] and PvDBP [46] in *P. vivax* Korean isolates. The dN was slightly greater than the dS in Pvs25, Pvs48/45, and PvWARP, suggesting the possible action of positive natural selection, but the differences were not significant.

**Table 3:** Estimates of DNA sequence polymorphism and tests of neutrality at TBV candidate antigens among Korean isolates

**Antigens**	**Total number of isolates**	**Segregating sites (S)**	**Singleton variable sites**	**Parsimony informative sites**	**Total no. of mutations**	***K***	**H**	**Hd ± SD**	**π ± SD**	**dN**	**dS**
Pvs25	86	1	0	1	1	0.427	2	0.427 ± 0.040	0.00065 ± 0.00006	0.00106	0.00000
Pvs28	86	0	0	0	0	0.000	1	0.000	0.00000	0.00000	0.00000
Pvs48/45	86	2	0	2	2	0.716	3	0.611 ± 0.026	0.00053 ± 0.00004	0.00069	0.00000
PvWARP	86	1	0	1	1	0.090	2	0.090 ± 0.041	0.00010 ± 0.00005	0.00020	0.00000

*K*, average number of pair-wise nucleotide differences; H, number of haplotypes; Hd, haplotype diversity; π, observed average pair-wise nucleotide diversity; dN, number of nonsynonymous substitutions; dS, number of synonymous substitutions.

## Conclusions

The results of the present study are in agreement with previous observations that TBV candidate antigens of *P. vivax* show highly limited sequence polymorphisms. No evidence for annual genetic variation of these antigens was also identified in Korean isolates. The limited genetic diversity of sexual stage antigens is most likely attributed to the expression of these proteins across mosquito stages, which might avoid immune pressure in the humans. The limited polymorphism observed in these TBV candidate antigens among *P. vivax* Korean isolates can provide useful baseline information for developing an effective TBV based on these antigens, for predicting the performance of the TBV, and to help identify polypeptide regions suitable for designing vaccines.

## Competing interests

The authors declare that they have no competing interests.

## Authors’ contributions

JMK, HLJ, SUM, and PYC performed all the experiments and analysed the sequence data. PYC, YKP, SHC and TSK collected the blood samples. BKN and TSK designed the study and supervised the study process. BKN wrote the paper. YYB, WMS, SHC, and TSK assisted in writing and editing the manuscript. All authors read and approved the final manuscript.
